# Factors associated with leisure-time physical activity among patients undergoing hemodialysis

**DOI:** 10.1186/s12882-015-0183-5

**Published:** 2015-11-27

**Authors:** Clara S. C. Rosa, Denise R. Bueno, Giovana D. Souza, Luís A. Gobbo, Ismael F. Freitas, Giorgos K. Sakkas, Henrique L. Monteiro

**Affiliations:** Unesp - Instituto de Biociências, Campus de Rio Claro, Seção Técnica de Pós-graduação, Sao Paulo State University, Avenida 24-A n° 1515 - Bairro Bela Vista, 13506-900 Rio Claro/SP, Brazil; Department of Public Health and Nutrition, University of Sao Paulo, Sao Paulo, Brazil; Department of Medicine, Sao Paulo State University, Botucatu, Brazil; Department of Physical Education, Sao Paulo State University, Presidente Prudente, Brazil; School of Physical Education and Sport Science, University of Thessaly, Trikala, Greece; Department of Physical Education, Sao Paulo State University, Bauru, Brazil

**Keywords:** Exercise, Physical activity, End-stage renal disease, Chronic kidney diseases, Barriers, Comorbidity

## Abstract

**Background:**

End-stage renal disease patients are characterized by low levels of physical activity, especially during leisure time. However, the recognition of variables associated with patterns of physical activity in this population has been little explored. Thus, the objective was to assess factors associated with levels of physical activity during leisure time among patients on haemodialysis.

**Methods:**

Ninety-eight patients (51.6 ± 15.7 years, 57 M/41 F) from two dialysis centres in São Paulo, Brazil participated in this cross-sectional study. Participants were divided into those who never exercised during leisure-time (inactive) and those who exercised at least once a week (active). The independent factors assessed were: socio-demographic data, comorbidities, personal barriers to exercise and physical activity records from childhood to adulthood (tracking of physical activity).

**Results:**

Only 27 % of patients were engaged in PA during their leisure time at least once a week. Patients who engaged in regular physical activity during adulthood before the initiation of the hemodialysis treatment (adjusted OR: 7.24 95 % IC: 1.99; 26.50), those who developed the renal disease through diseases other than diabetes or hypertension (adjusted OR: 4.82; 95 % IC: 1.48; 15.68), and those who had no cardiovascular diseases (adjusted OR: 11.33; 95 % IC: 1.23; 103.8) where more likely to be active during their leisure-time.

**Conclusion:**

Comorbidities such as cardiovascular disease, hypertension and diabetes mellitus as well as the level of physical activity prior to end-stage renal disease could predict leisure-time physical activity among patients receiving hemodialysis therapy.

## Background

End-stage renal disease (ESRD) patients are characterized by severe functional limitations such as low cardiorespiratory fitness, fatigue, muscle atrophy, malnutrition, and other health problems, all of which are linked to reduced survival [1]. Additionally, these patients suffer from associated chronic conditions including hypertension, coronary artery disease, type II diabetes, and depression. Moreover, ESRD patients experience impaired physical performance, which, directly affects functional capacity and overall health related quality of life [2].

The recognition of exercise as a safe and effective rehabilitation program has become increasingly evident. Recent studies have gathered evidence that exercise can improve cardiovascular fitness [3], functional capacity [4], muscular strength [5], muscle atrophy [6] and reduce some risk factors related to cardiovascular diseases [7], as well as contribute to improving the quality of life and survival of these patients [8]. However, recent studies have shown that ESRD patients, especially the elderly, reporting low levels of physical activity (PA) and overall functional capacity [9, 10].

Despite public health recommendations for PA, as it is accumulated in various domains of PA [11], such as occupational, transportation, and household, the domain of leisure-time PA has been extensively studied, and it shows consistent association with risk factors for cardiovascular disease and survival among the general population [12] and patients suffering from chronic kidney disease (CKD) [13–16].

These studies demonstrate that inactive behaviour can be determined by socio-demographic factors such as years of education and ethnicity as well as various environmental factors [17–19]. In addition, PA practiced during youth has been studied as a possible predictor of PA in adulthood life, and associated with a lower occurrence of chronic diseases such as dyslipidaemia, hypertension and diabetes in adulthood [20, 21].

However, in the ESRD population, despite the evidence showing that low PA is a significant risk factor for higher morbidity and lower survival, the recognition of variables associated with the habit of regular PA has been insufficiently explored. Information about PA patterns in ESRD patients is important not only to identify those who need physical rehabilitation, but also to identify factors responsible for low adherence to any PA or to various exercise intervention programs. Therefore, the aim of the current study was to analyse factors associated with levels of PA during leisure time in ESRD patients on haemodialysis (HD) therapy.

## Methods

### Subjects

The current study included patients from two HD centres in São Paulo, Brazil. Each unit provides six shifts of HD sessions with about 25 patients per shift. Three shifts from each hospital were selected. From hospital 1 we selected the afternoon shift for the Monday–Wednesday–Friday group and, afternoon and evening shifts for the Tuesday–Thursday–Saturday group. From hospital 2 we selected morning and afternoon shifts for the Monday–Wednesday–Friday group and the evening shift for the Tuesday–Thursday–Saturday group. The inclusion criteria were: a) to be over 18 years of age; b) to have been on HD therapy for more than three months; c) to be able to reply to questionnaires and d) to have spontaneously participated in the study. Patients unable to walk were excluded from the sample.

This cross-sectional study was approved by the research ethics committee of the Sao Paulo State University, Faculdade de Ciências – Bauru Campus (Case No. 1048/46/01/10) and written informed consent was obtained from all participants before the study began.

### Physical activity levels during leisure-time

Patients were asked if they had performed any kind of regular PA at least once a week for the previous month. Based on the levels of PA, they were grouped into patients who were active at least once a week – “Active Group” (reference); and patients who were not active during their leisure time –“Inactive Group” (contrast).

### Leisure-time PA validation

In a subsample of 40 patients, the agreement between the questionnaire used and the direct measurement of PA was assessed by an Actigraph System (Actigraph GT3X, Actigraph LLC, Pensacola, FL). Accelerometers were placed on the patients’ waists by using an elastic band. The participants wore the accelerometer for eight days (one full week). They were instructed to wear the accelerometer all day long except for water-based activities, such as personal hygiene or swimming, and during sleep. ActiLife5 Data Analysis Software by Actigraph was used for the data analysis. The epoch was set at 60-s as in this population PA is characterized by low intensity and long duration, which is standard for monitoring free-living physical activity in adults. Data with periods of continuous zero values for more than 60 min were taken as the participant having removed the accelerometer. At least 5 days of recording with a minimum of 10 h of registration per day were necessary for the patient to be included in the study. The time spent in moderate-vigorous PA per week was determined by counts ≥1952 per minute.

Patients were classified by time per week spent in moderate-vigorous PA: <150 min/week and ≥150 min/week. Next, to analyse the agreement between leisure-time PA and accelerometer in identifying subjects that reached ≥150 min/week, the Kappa index was tested. The ROC curve and its parameters (sensibility and specificity) were calculated in order to classify individuals according to the global standard guideline for sufficient PA of ≥150 min/week of moderate-vigorous PA.

A good agreement with the motion sensor accelerometer was found (*Actigraph* GT3X) (Kappa index = 0.48, *p* = 0.001), in addition, high sensitivity (88.5 %) and moderate specificity (42.9 %), were used to identify those who performed moderate or vigorous activity equal to or greater than 150 min per week.

### Epidemiological variables

The variables obtained through interviews were categorized as follows: age (<60 years and ≥60 years); skin colour (white/others and black); employment status (employed/housewives and retired/unemployed); and years of education (<8 years and ≥8 years).

Clinical measurements were assessed from patients’ clinical records and categorized as follows: ESRD aetiology (hypertension/diabetes mellitus and other causes), HD vintage (≤3 years and >3 years), and other morbidities which were categorized into present or absent according to the International Statistical Classification of Diseases and Related Health Problems 10^th^ Revision (ICD-10 block): endocrine, nutritional and metabolic diseases (E00–E90); circulatory system disease (I00–I99); and the musculoskeletal system and connective tissue diseases (M00–M99).

### Questionnaires

Personal barriers to exercising in leisure-time were also analysed through a questionnaire developed by Reichert et al. (2007) [19], and composed of eight closed questions: 1) dislike exercise, 2) feel too tired, 3) feel too old, 4) fear of injury, 5) lack of time, 6) lack of company, 7) having an injury/disease, and 8) lack of money, was adapted and two questions were added as follows: 9) “Is chronic kidney disease a barrier for exercising?” and, 10) “Is haemodialysis treatment a barrier for exercising?”. For the purposes of the current analysis, a negative and a positive response were considered.

The habit of exercise in different periods of life, from childhood (7–17 years) to adulthood (before the initiation of the HD treatment) were analysed based on the subjects’ recollections. Activities performed at gyms, and at school were included in the assessment. Engaging in PA without supervision was also taken into account. Only activities performed for at least six consecutive months were included.

### Statistical analysis

Sample characteristics were presented in mean and standard deviation, and normally distributed (Kolmolgorov-Smirnov test), while categorical variables were described as absolute values and percentages.

The chi-square (*χ*^2^) evaluated possible associations between dependent and independent variables. Therefore, a binary logistic regression (*forward stepwise*) was developed with the associations that showed statistical significance up to 20 % (*p* <0.20) and with age, gender and race. All analyses were performed using SPSS version 13.0 with a significance set at *p* ≤0.05.

## Results

Ninety-eight out of the 100 participating patients completed the interview and presented all available data records. The average age was 51.6 ± 15.7 years (range from 20 to 89 years) and HD vintage was 48.3 ± 41.8 months (range 3–167 months). The proportion of active patients during leisure-time was 27.6 %. The main type of activity reported by the majority of patients was walking; one patient reported exercising in a gym and two others played football (soccer). The average age in the two groups in Table 1 were: inactive group, 52.16 ± 15.69 and active group, 50.27 ± 15.82 (*p* = 0.834).

**Table 1:** Leisure-time physical activity according to socio-demographic variables, hemodialysis vintage, ESRD etiology and morbidities (*n* = 98)

	Leisure-time physical activity			*X* ^2^
	Total *N* (%)	Active group *N* (%)	Inactive group *N* (%)	*p*-value
Sex				
Male	57 (58.2)	17 (29.8)	40 (70.2)	0.639
Female	41 (41.8)	10 (24.4)	31 (75.6)	
Age				
<60 years	71 (72.4)	20 (28.2)	51 (71.8)	0.824
≥60 years	27 (27.6)	7 (25.9)	20 (74.1)	
Skin color				
White/other	59 (60.2)	14 (23.3)	46 (76.7)	0.298
Black	39 (39.8)	13 (34.2)	25 (65.8)	
Work occupation				
Working/house-wife	46 (46.9)	13 (28.3)	33 (71.7)	0.882
Retired/unemployed	52 (53.1)	14 (26.9)	38 (73.1)	
Studying years				
<8 years	62 (63.3)	10 (22.2)	35 (77.8)	0.191*
≥8 years	36 (36.7)	17 (32.1)	36 (67.9)	
Hemodialysis vintage				
≥3 years	46 (46.9)	16 (34.8)	30 (65.2)	0.132*
<3 years	52 (53.1)	11 (21.2)	41 (78.8)	
ESRD etiology				
Hypertension/Diabetes	55 (56.1)	9 (16.4)	46 (83.6)	0.005**
Other disease	43 (43.9)	18 (41.9)	25 (58.1)	
Morbidity				
Metabolic				
Yes	36 (36.7)	11 (30.6)	25 (69.4)	0.612
No	62 (63.3)	16 (25.8)	46 (74.2)	
Cardiovascular^a^				
Yes	92 (93.9)	23 (25.0)	69 (75.0)	0.047**
No	6 (6.1)	4 (66.7)	2 (33.3)	
Musculoskeletal^a^				
Yes	16 (16.3)	1 (6.3)	15 (93.8)	0.062*
No	82 (83.7)	26 (31.7)	56 (68.3)	

Nota. **p < 0.05; * *p* < 0.20; ^a^ fisher exact test

Table 1 describes the various factors associated with leisure-time PA levels in ESRD patients. HD vintage and amount (years) of education showed an association with *p* < 0.20 in leisure-time PA. Patients on HD for more than 3 years as well as those with more years of education were more likely to be adequately active.

Hypertension was the predominant ESRD ethology reported (39.8 %), followed by diabetes mellitus (16.3 %). Interstitial nephritis and polycystic kidney disease were observed in 15.3 and 14.3 % of the patients, respectively, while, pyelonephritis (4,1 %) and unknown factors (10.2 %) composed the rest of the causes. Patients with “other diseases” for their ESRD primary aetiology showed a higher prevalence of active life style (49.1 % vs. 16.4 %; *p* = 0.005) compared to their counterparts.

Circulatory and musculoskeletal system diseases were associated with levels of PA during leisure-time; patient who did not report cardiovascular disease (66.7 % vs. 25.0 %; *p* = 0.047), and musculoskeletal disease (31.7 % *vs.* 6.3 %; *p* = 0.062) were more likely to be adequately active compared to their counterparts.

Personal barriers and its associations with leisure-time PA were described in Table 2. Feeling too tired, followed by CKD and haemodialysis therapy were the most common reported barriers. However, only the “disliking exercise” reason showed an association of *p* <0.20 in the univariate analysis.

**Table 2:** Perceived barriers and current leisure-time physical activity

		Leisure-time physical activity		*p*
	Total *N* (%)	Yes (%)	No (%)	
Feel too tired	62 (63.3)	59.3	64.8	0.612
Chronic kidney disease	50 (51.0)	51.9	50.7	0.919
Hemodialysis treatment	47 (48.0)	44.4	49.3	0.821
Lack of time	39 (39.8)	33.3	42.3	0.493
Lack of company	37 (37.8)	29.6	40.8	0.357
Lack of money	36 (36.7)	44.4	33.8	0.329
Fear of injury	35 (35.7)	40.7	33.8	0.522
Disliking exercise	25 (25.5)	12.0	88.0	0.068*
Feel too old	18 (18.4)	14.8	19.7	0.772
Having an injury/disease	14 (14.3)	11.1	15.5	0.752

Note. *p < 0,20

Regular PA during childhood and adolescent years was 30.6, and 48 % respectively. However, only 16.3 % practiced regular exercise in both phases; in addition, from these patients, only 9.2 % still remained active. During childhood and adolescence, the dominant sports activities were football, basketball, volleyball and swimming while during adulthood, the most common activities were walking, working out at the gym, football and dancing.

**Fig. 1:**
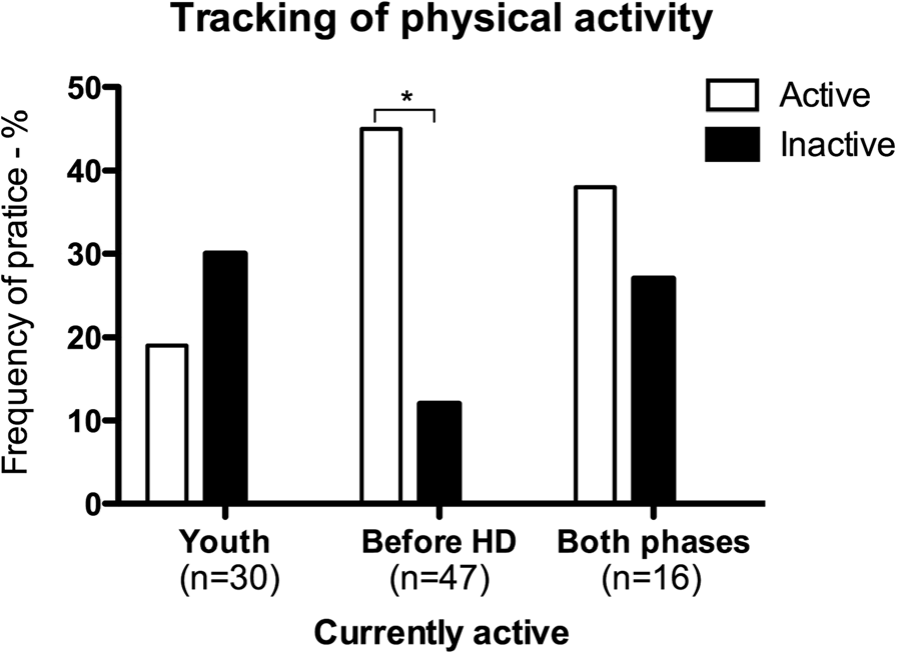
Association between current active habits during leisure time and habits of exercising during childhood (youth) and adulthood before the initiation of hemodialysis (before HD) and combined (both phases); *p < 0.005 in the chi-square test

Patients who were engaged in regular PA before the initiation of the haemodialysis therapy were more likely to be adequately active (44.7 % vs. 11.8 %; *p* = 0.005) (Fig. 1).

In the multiple analysis (Table 3), the magnitude of association, adjusted for potential confounders, showed that the habit of being active before the start of renal replacement therapy resulted in a seven times greater chance of being active during leisure time compared to inactive patients. In addition, the lack of diabetes mellitus or hypertension as a cause of ESRD as well as the absence of circulatory diseases leads to 4.8 and 11 more time being active, respectively.

**Table 3:** Final model of logistics and their respective values of OR, confidence interval (95 % CI) and statistical significance (*p*) between physical activity in leisure and independent variable regressions

	Active in leisure-time physical activity		
	OR*	95 % CI	*P*
Before starting HD therapy			
Active	7.24	1.99–26.50	0.003
Inactive	1		
Cardiovascular morbidity			
Yes	1		
No	11.33	1.23–103.80	0.032
ESRD etiology			
Hypertension/Diabetes	1		
Other disease	4.82	1.48–15.68	0.009

Multiple logistic regressions. Odds ratio (OR) adjusted for age, gender, race, hemodialysis vintage, musculoskeletal diseases, years of education and the “disliking exercise” barrier

*Hosmer-Lemeshow test: *p* = 0.508. R^2^ = 0.44

Other disease, other disease for end stage renal disease etiology

## Discussion

The current findings indicate that cardiovascular diseases, the pre-existence of hypertension and diabetes mellitus as well as physical activity levels in the previous stages of CKD before the initiation of the haemodialysis therapy are strong predictors of the practice of leisure-time physical activity.

Studies indicate that ESRD patients on HD have low engagement in PA compared to the healthy population [9, 10, 22]. Corroborating this data, in our study only 26.7 % of participants were classified as sufficiently active, participating in exercise activities once or more than once/week during leisure time. However, this prevalence was almost half of those reported across the 12 DOPPS countries [15] which reached 47.4 % of the assessed patients. Such findings are important to identify patients with insufficient patterns of PA, in order to guide effective strategies aimed at increasing the level of physical capacity in this population, as well as to identify possible factors and barriers related to engagement in PA habits.

Active behaviour could be determined by numerous factors, and therefore it is important to differentiate the reason why patients in haemodialysis have low engagement in PA compared to other patients with chronic diseases or the general population. Certainly, HD treatment *per se* contributes to low participation in physical activities, since these patients, due to dialysis procedure, spend at least 12 h a week in a state of bedrest (during haemodialysis treatment) and report high levels of physical fatigue, especially after the HD treatment session [23], which contributes to a sedentary life [24]. Yet, although active habits may contribute to an improvement of several health indicators in ESRD, it is certain that the high degree of comorbidity associated with the ESRD itself could affect levels of physical inactivity leading to the vicious circle of a sedentary life style.

Similarly to our results, Stack et al. [25] found that chronic renal failure patients with history of cardiovascular disease or abnormal serum phosphorus levels (a variable associated with elevated risk for cardiovascular disease), had low engagement in regular physical exercise. From an epidemiological standpoint, this data is alarming, since, in HD patients, acute cardiovascular events affect about 9 % of this patients, which means an odds of 10 to 20 times higher than those observed in the general population [26].

Despite the benefits of cardiac rehabilitation programs in the various aspects of health (physical, mental and social), as well as a lower risk of cardiac death [27], the current literature had shown that many eligible patients are not referred to these programs and others are not encouraged to participate [28, 29]. Consequently, it is possible that the decreased cardiovascular health and capacity of these patients could contribute directly to a sedentary lifestyle, away from PA and active habits, leading inevitably to low functional capacity. Indeed in ESRD patients the levels of fitness in terms of VO_2_ values have been found to be as low as 15 to 25 ml/kg/min implying a disabled or severely debilitated mobility status [30, 31].

Nevertheless, hypertension and diabetes as a cause of CKD play a pivotal role in survival and quality of life in these patients. Diabetes and hypertension are the main causes of CKD in Brazil affecting 35 and 30 %, respectively [32] while the low level of PA is considered the major risk factor for both chronic morbidities which are developed over the years [33]. Thus, it is persuasive that the association between low levels of PA and the pre-existence of these morbidities in this segment of the ESRD patients is also a reflection of pre-existing inactive habits.

Even though age has been shown to affect the levels of PA in ESRD patients [10, 34, 35], our data did not support those finding. A possible explanation could be that these studies took into account the total PA and not only that performed during leisure time. It is likely that younger patients, despite haemodialysis therapy, maintain their occupational activities, resulting in higher levels of PA in relation to older people and retirees. Indeed 47 % of our patients reported some kind of occupation during weekdays, however our results showed no association between occupational status and leisure-time PA, as it has been shown in the non-kidney-disease population [36]. On the other hand, and similar to our results, education levels showed an association to PA in leisure time, and an inverse association with occupational and household activities [36].

Perceived barriers have also been studied in the general population and it is demonstrated that the environmental perception or personal barriers are inversely associated with PA level [19]. In chronic renal populations, these barriers may also act as determinants of PA [37]. Contrariwise, our study showed a close association only between the barrier of “dislike exercise” and leisure-time PA. However, studies show that the type of barriers reported, as well as the magnitude of the association with PA, varies from population to population [38]. For example, the barrier “lack of money” was reported by almost 40 % of our sample, in contrast to those found in the literature [37, 39]. This is not surprising, since the studies cited previously represent samples from developed countries. Thus, these results emphasize the need for more studies in populations representing different geographical and socio-economical statuses.

In addition to the factors cited above, evidence shows that many factors that influence the health of adults starts at a young age, such as chronic diseases and active lifestyle habits [20, 40]. Our results have demonstrated that ESRD patients are more likely to remain active when they maintain active habits at an early stage of CKD. It seems that when the benefits of regular PA are well-established at an early age, it influences the future phases of a persons’ life [41]. It is also worth restating that an early referral to a nephrologist and the work of a multidisciplinary team in the care of pre-dialysis patients in order to implement pre-dialysis care and successful promotion of physical activity and exercise is very important [42].

To our knowledge, our study is the first to report data from leisure-time PA of ESRD patients in Brazil as well as the first in exploring factors associated with this behaviour, such as the tracking of PA levels. Thus, more studies are needed to improve the conclusions on this subject for ESRD, since the cross-sectional characteristics of this study compromise the data inference. In addition, other potential factors that may influence PA, such as nutritional status and muscle impairment [6, 43] could not be evaluated in this study. Furthermore, our results emphasize the need for more studies regarding physical activity patterns among early-stage transplant and CDK patients (stages 1–4), as well as additional information regarding patients on automated and continuous ambulatory peritoneal dialysis. These additional studies would provide complete data for better conclusions.

The recognition of low levels of PA related to CKD in the haemodialysis population has gained recognition in the literature. However, the promotion of this active habit in this population is still infrequent. The knowledge generated through the information about the PA levels among haemodialysis patients are important in addressing the factors responsible for poor adherence, as well as identifying risk groups in need of physical rehabilitation or other interventions in order to focus on developing strategies for increasing the habit of PA in this population.

## Conclusion

It is likely that the low level of physical activity in leisure time found in this group of ESRD patients undergoing HD therapy is associated, for the most part, with pre-existing habits. Consequently, attention is drawn to strategies promoting active habits at an early stage of CKD (stages 1–4), especially in those with the presence of chronic hypertension and diabetes. A greater effort to encourage rehabilitation regimens in this high-risk group of patients by reintegrating their social activities is required in order to improve their ability to exercise and to continue to benefit from PA programs. Thus, it is important to promote the engagement of health professionals focused on physical rehabilitation as a necessary part of any multidisciplinary renal team.

## Abbreviations

ESRD: end-stage renal disease
PA: physical activity
CKD: chronic kidney disease
HD: hemodialysis
